# Enhanced Cd Phytoextraction by *Solanum nigrum* L. from Contaminated Soils Combined with the Application of N Fertilizers and Double Harvests

**DOI:** 10.3390/toxics10050266

**Published:** 2022-05-19

**Authors:** Wei Yang, Huiping Dai, Lidia Skuza, Shuhe Wei

**Affiliations:** 1 Pollution Ecology and Environmental Engineering, Institute of Applied Ecology, Chinese Academy of Sciences, Shenyang 110016, China; 937364967@qq.com; 2 Shaanxi Province Key Laboratory of Bio-Resources, College of Biological Science and Engineering, Shaanxi University of Technology, Hanzhong 723001, China; 3 Institute of Biology, University of Szczecin, 71-415 Szczecin, Poland; lidia.skuza@usz.edu.pl

**Keywords:** hyperaccumulator, phytoremediation, Cd-contaminated soil, exogenous additives, antioxidant enzyme activity

## Abstract

It is very important to increase phytoremediation efficiency in practice in suitable climatic conditions for plant growth through multiple harvests. *Solanum nigrum* L. is a Cd hyperaccumulator. In the present experiment, after applying different types of N fertilizers (NH_4_HCO_3_, NH_4_Cl, (NH_4_)_2_SO_4_, CH_4_N_2_O), root and shoot biomasses and Cd phytoextraction efficiency of *S. nigrum* effectively improved (*p* < 0.05). Shoot biomasses of *S. nigrum* harvested at the first florescence stage plus the amounts at the second florescence stage were higher than those harvested at the maturation stage, which indicates that *S. nigrum* Cd phytoaccumulation efficiency was higher in the former compared to the latter as there was no clear change in Cd concentration (*p* < 0.05). The pH value and extractable Cd contents showed no changes, regardless of whether N fertilizer was added or not at different growth stages. In addition, after N fertilizer was applied, H_2_O_2_ and malondialdehyde (MDA) contents in *S. nigrum* in vivo were lower compared to those that had not received N addition (CK); similarly, the concentration of proline was decreased as well (*p* < 0.05). The activity of the antioxidant enzyme catalase (CAT), harvested at different growth periods after four types of N fertilizer applications, obviously decreased in *S. nigrum* shoots, while peroxidase (POD) and superoxide dismutase) (SOD) activities increased (*p* < 0.05). Our study demonstrated that (NH_4_)_2_SO_4_ treatment exerted the most positive effect and CH_4_N_2_O the second most positive effect on *S. nigrum* Cd phytoremediation efficiency in double harvests at florescence stages, and the growth conditions were better than others.

## 1. Introduction

Cadmium (Cd), as one of the most poisonous heavy metals in the environment, has extremely toxic biochemical characteristics, even at low levels [1], seriously threatening the growth, development and yields of crops and causing a series of damages at morphological, physiological and biochemical levels in plant cells and tissues, even harming human health when ingested [2]. Phytoremediation, a novel biological technique that utilizes plant materials—hyperaccumulators to remediate soil contaminated by heavy metals—is superior to many other physical and chemical measures due to its high efficiency, environmental friendliness, long-term availability and low price [3,4]. Usually, hyperaccumulators show three basic characteristics. Firstly, heavy metals generally have a higher concentration in plants than in soils; secondly, the average shoot concentration of Zn is 10,000 DW mg∙kg^−1^, while that of Cu, Pb, Ni and Co is 1000 DW mg∙kg^−1^, and that of Cd is 100 DW mg∙kg^−1^; thirdly, the accumulation concentration of heavy metals in the aboveground parts is higher than in the root, while the plants grow well and no obviously toxic symptoms appear [2]. Some hyperaccumulators include Cd hyperaccumulators *Noccaea caerulescens* [5], *Solanum nigrum* L. [6] and *Viola baoshanensis* [7], Cr hyperaccumulator *Leersia hexandra* Swartz [8], Zn hyperaccumulator *Sedum alfredii* Hance [9,10], Ni hyperaccumulator *Alyssum murale* [11] and As hyperaccumulator *Pteris vittata* [12,13]. However, for most hyperaccumulators, the disadvantages of slow growth, a low-weight yield and geographical constraints limit their potential to amend large-scale heavy-metals-polluted arable land [14], and thus they cannot achieve better remediation efficiency in practice. The addition of environmentally friendly additives, such as inorganic, organic fertilizers and bio-fertilizers [15], as well as CO_2_ gas supply [16] and intercropping [17], could improve phytoextraction efficiency by hyper- and intermediate accumulators owing to the increased biomasses and/or the improved Cd shoot concentration.

Nitrogen (N) is an essential major element that improves soil fertility and biochemical properties and increases crop biomass yields (it is involved in many metabolic processes, including the biosynthesis of amino acids, nucleic acids and proteins in plants, and improves the resistance to abiotic and biotic stresses. For non-hyperaccumulators, most research indicates that N application reduces the production of a large number of reactive oxygen species (ROSs) induced in vivo by biotic and abiotic stresses, such as superoxide radicals (O_2_^−^), hydrogen peroxide (H_2_O_2_) and hydroxyl radicals (OH), and maintains the balance of the generation and elimination of ROSs so as to withstand the secondary oxidative stress [18]. Simultaneously, it generates a synergistic effect on sophisticated antioxidant defense systems to increase the contents of low-molecular-weight non-enzymatic antioxidants, such as proline, GSH and AsA, and improves the activities of antioxidant enzymes, including superoxide dismutase (SOD), peroxidase (POD), catalase (CAT) and another four enzymes in the *AsA*-*GSH* recycle system composed of ascorbic acid peroxidase (APX), glutathione reductase (GR), dehydroascorbic acid reductase (DHAR) and monodehydroascorbic acid reductase (MDAR) to alleviate Cd toxicity in plants, thereby protecting the plant itself [19]. These biochemical indexes are generally recognized as the most sensitive and typical parameters in evaluating plant tolerance to biotic and abiotic stresses [20,21].

Previous articles pointed out that N fertilizer could mitigate the adverse effect of Cd and decrease Cd uptake through non-hyperaccumulators. Conversely, some studies of N application showed that N fertilizer supply could enhance phytoremediation of Cd-contaminated soils by increasing the shoot biomass yields and not decreasing Cd uptake through hyperaccumulators [22,23]. Furthermore, other studies discovered N could mitigate damages caused by leaf reduction and senescence due to its influence on cell division at different growth stages [24,25]. Generally, N application has different effects on Cd bioaccumulation and antioxidant defense mechanisms under biotic and abiotic stresses between hyperaccumulators and non-hyperaccumulators.

Moreover, previous studies revealed that multiple cropping harvests in a year at its flowering stage could shorten the phytoremediation period in Cd-polluted soil due to the enhanced Cd phytoextraction rate, therefore improving the Cd phytoremediation efficiency significantly [26,27]. Currently, there is little detailed research in the literature reporting the effect of N fertilizers on Cd phytoextraction and antioxidants and antioxidant enzymes through the Cd hyperaccumulator *S. nigrum* and comparing single harvests with double-cropping harvests over one year.

Taking into consideration the preceding results, the newly found Cd hyperaccumulator *S. nigrum* was selected as the present experiment material. It has attracted widespread attention due to its distinctive advantages of a rapid growth rate, viability and environmental suitability [22], and the objective of our study was to investigate which fertilizers would obtain maximum Cd phytoaccumulation in different growth stages and induce the strongest antioxidant enzyme activities so as to maintain the available growth stage under different types of N fertilizers. In addition, compared to single-cropping harvest, double-cropping harvests achieved better optimization of Cd removal from contaminated soil.

## 2. Materials and Methods

### 2.1. Basic Physicochemical Properties of Soil and the Pot Experiment

The used soil sample was collected from a local agricultural field with meadow-brown soil, and the soil sample was taken from neutral soils. Seeds of *S. nigrum* were also collected from the local field. The soil properties and local climate conditions are all consistent with the previous article (detailed data are omitted due to Crossref Similarity Check) [23].

Distilled water was spiked with Cd with no Cd detected in the form of CdCl_2_∙2.5H_2_O, and the Cd concentration in all treatment groups was 2 mg∙kg^−1^. Compared with the Soil Environmental Quality Standard in China (GB-15618-2018), the pollution level was moderate [28]. The pure nitrogen fertilizers used (NH_4_HCO_3,_ NH_4_Cl, (NH_4_)_2_SO_4_ and CH_4_N_2_O) were purchased at the local market and added to Cd. The detailed experimental design is shown in Table 1. Soil samples (2.5 kg of dry mass) were put in plastic pots and equilibrated for 2 months.

Six *S. nigrum* seedlings with four leaves and of a uniform height were transplanted from the seedling tray into each pot, and each treatment was repeated 3 times. Plants were irrigated with tap water twice per day to maintain approximately 80% soil moisture content. Single-cropped plants were harvested at maturity, while double-cropped plants were harvested at the first florescence stage (Table 1), i.e., the first round of harvest at the florescence stage (the first florescence stage), and then *S. nigrum* seedlings were transplanted until harvest at the florescence stage (the second florescence stage). The maturation stage (118 days) was roughly equal to the time it took for seedlings to evolve from the first florescence stage (59 days) to the second florescence stage (59 days) in one year.

### 2.2. Chemical Analysis

Plant biomass and Cd concentration were measured according to Yang et al. (2019) [23]. In brief, the plant samples were divided into roots and shoots, and dried in the oven. The available Cd in the soil was extracted with 1 mM MgCl_2._ Cd concentrations in plant and soil samples were digested using concentrated HNO_3_ and HClO_4_, and determined by AAS (Hitachi 180, Hitachi Co., Ltd., Shanghai, China). The certified standard reference material (NIST SRM 1547, peach leaves, Guangzhou Jike Instrument Technology Co., Ltd., Guangzhou, China) was used for quality control. Likewise, organic matter, nitrogen and extractable P contents were measured using a method described by Lu (2000) [29]. The pH value was determined by a pH meter (PHS-3B, Shanghai Puzhen Biological Technology Co., Ltd., Shanghai, China), and the soil-to-water ratio was 1:2.5 (*v*/*w*) [22].

### 2.3. Analysis of Enzyme Activity

Malondialdehyde (MDA) content, which is was used as a parameter for the evaluation of lipid peroxidation, was measured as the degree of damage to the plants under abiotic or biotic stress. In this experiment, MDA concentration was determined as described by Zhu et al. (2019) [30]. In brief, fresh tissues (0.5 g) were homogenized in a mortar containing 5 mL of 10% trichloroacetic acid (*w*/*v*) (TCA) with 0.25% (*w*/*v*) 2-thiobarbituric acid (TBA). The mixture was heated at 95 °C for 30 min, and then quickly cooled in an ice bath and centrifuged at 10,000 rpm for 10 min. The data of the supernatant were measured at 532 nm. H_2_O_2_ content was measured using the previous method [31]. Briefly, fresh tissues (0.5 g) were firstly ground in 5 mL of 50 mM sodium phosphate buffer (pH 7.8), and then the mixture was centrifuged at 12,000 rpm for 10 min under 4 °C. The values of supernatants of TiCl_4_ added to 20% H_2_SO_4_ (*v*/*v*) were obtained at 410 nm after 5 min at room temperature.

The proline content was determined according to Bates et al. (1973) [32]. Fresh tissues (0.5 g) were firstly ground in 5 mL of 3% sulfosalicylic acid, and then the homogenate was centrifuged at 3000 rpm for 5 min. After light-proofing treatment one hour later, the 2 mL supernatant was transferred into a large test tube containing 2 mL of glacial acetic acid and 2 mL of acidic ninhydrin; subsequently, the mixture was heated in a boiling-water bath for 20 min, and then the cooled filtrate was measured at 520 nm.

CAT activity was measured by a decrease revealed at 240 nm absorbance for 3 min in presence of H_2_O_2_, which is a modified method described by Hasanuzzaman et al. (2011) [33]. A total of 0.5 g of fresh tissues was homogenized in 3 mL of 50 mmol L^−1^ phosphate buffer, pH 7.8, containing 1.0 mmol L^−1^ EDTA and 2% (*w*/*v*) polyvinylpyrollidone (PVP). Then, the homogenate was centrifuged at 5000× *g* for 10 min at 4 °C, and the supernatant was used for the enzymatic assays. Superoxide dismutase (SOD) and peroxidase (POD) activities were determined according to the provided references [34]. One unit of SOD (U) was considered the amount of enzyme needed to inhibit 50% of reduction in nitro blue tetrazolium (NBT) revealed at 560 nm. The values of POD activities were determined by the guaiacol oxidation rate of H_2_O_2_ consumption at 470 nm absorbance. All treatments were performed with three replicates.

### 2.4. Data Processing and Statistical Analysis

Data processing and summary statistics were performed using Microsoft Excel 2000 (IBM, Manhattan, NY, USA). Means of different treatments were compared using one-way ANOVA and two-way ANOVA with DPS software (Hangzhou, China). For post hoc test, LSD multiple comparison was performed, and the significance level was *p* < 0.05 based on the assumption of normal distribution and homogeneity of variance [35].

## 3. Results

### 3.1. Effects of Different Types of N Fertilizers on Shoot Phytoextraction of Cd in S. nigrum

As seen in Table 2, under single and double harvests of *S. nigrum*, was no significant change in Cd concentration in roots and aboveground parts (shoots) of *S. nigrum* after applying different fertilizers (NH_4_HCO_3_, NH_4_Cl, (NH_4_)_2_SO_4_ and CH_4_N_2_O) compared to CK with no fertilizer addition (*p* < 0.05).

Nevertheless, the analysis of the single- and double-harvest experiments showed that Cd phytoremediation efficiency (μg plant^−1^) in the shoots after application of four types of nitrogen fertilizers (NH_4_HCO_3_, NH_4_Cl, (NH_4_)_2_SO_4_ and CH_4_N_2_O) significantly increased compared to CK (*p* < 0.05). When *S. nigrum* was harvested at the maturation stage, N additions significantly increased Cd phytoremediation efficiency compared to the CK (17.66 μg plant^−1^): NH_4_HCO_3_ increased by 1.20-fold (F3), NH_4_Cl increased by 1.14-fold (F6), (NH_4_)_2_SO_4_ increased by 1.88-fold (F9), and CH_4_N_2_O increased by 1.84-fold (F12), respectively.

When *S. nigrum* was harvested at the first and the second florescence stages, the Cd phytoremediation efficiency (μg plant^−1^) in the shoots was basically the same (F2 and F3, F5 and F6, F8 and F9, F11 and F12), and their averages were 24.16 μg plant^−1^, 25.61 μg plant^−1^, 34.01 μg plant^−1^ and 33.03 μg plant^−1^, i.e., significantly increased by 36.8%, 45.0%, 92.6% and 87.0%, respectively, compared to the CK.

In one year, the Cd phytoremediation efficiency (μg plant^−1^) in the shoots at the first florescence stages plus the second florescence stages (double harvest) were 48.32 μg plant^−1^ (F1+F2), 51.31 μg plant^−1^ (F4+F5), 68.02 μg plant^−1^ (F7+F8) and 66.60 μg plant^−1^ (F10+F11), i.e., significantly increased by 1.74-fold (NH_4_HCO_3_), 1.90-fold (NH_4_Cl), 2.85-fold ((NH_4_)_2_SO_4_) and 2.77-fold (CH_4_N_2_O) compared to the CK, respectively. Obviously, the Cd phytoremediation efficiency (μg plant^−1^) in the shoots of double-harvested plants increased by 24.3% (F1+F2 vs. F3), 35.7% (F4+F5 vs. F6), 33.8% (F7+F8 vs. F9) and 32.3% (F10+F11 vs. F12), respectively.

### 3.2. Effects of Different Types of N Fertilizers on Root and Shoot Biomasses in S. nigrum

As shown in Table 3, dry weights of roots and aboveground plant parts of *S. nigrum* markedly increased (*p* < 0.05) in all treatment groups after applying the same amount of the four types of N fertilizers (NH_4_HCO_3,_ NH_4_Cl, (NH_4_)_2_SO_4_ and urea (CH_4_N_2_O)); dry weights of roots and aboveground parts were: 0.41 g plant^−1^, 1.84 g plant^−1^, 0.42 g plant^−1^, 1.86 g plant^−1^, 0.69 g plant^−1^, 2.41 g plant^−1^ and 0.70 g plant^−1^ and 2.41 g plant^−1^, respectively, and in comparison to CK, they were increased by 1.64-, 1.15-, 1.66-, 1.18-, 3.43-, 1.81- and 3.47- and 1.81-fold, respectively (*p* < 0.05). Using four types of N fertilizer (NH_4_HCO_3_, NH_4_Cl, (NH_4_)_2_SO_4_ and urea (CH_4_N_2_O)) treatments, our study confirmed that root and shoot biomasses harvested at the mature stage were higher than those harvested at the first and second florescence stages. In addition, it was calculated that total dry biomasses (g plant^−1^) of the aboveground *S. nigrum* parts harvested at the first and second florescence stages with the double-cropping method over the course of one year were much larger than those harvested at the maturation stage with the single-cropping method over one year; of all treatments, the (NH_4_)_2_SO_4_ and urea (CH_4_N_2_O) treatment groups obtained the highest shoot biomasses, and urea (CH_4_N_2_O) treatment showed the second highest (Table 3), i.e., the change trends of shoot biomasses were the same as those of the Cd phytoremediation efficiency (μg∙plant^−1^) (Table 2).

### 3.3. Effects of Different Types of N Fertilizers on H_2_O_2_ and MDA Contents in S. nigrum Shoots

Table 4 and Table 5 show that compared to CK without the addition of fertilizers, H_2_O_2_ (mg g^−1^) and MDA (μmol g^−1^) contents in *S. nigrum* shoots with four types of N fertilizer (NH_4_HCO_3,_ NH_4_Cl, (NH_4_)_2_SO_4_ and urea (CH_4_N_2_O)) markedly decreased (*p* < 0.05); at the maturation stage, H_2_O_2_ contents (mg g^−1^) decreased by 16.55%, 17.27%, 20.14% and 17.99%, respectively (*p* < 0.05). Similarly, MDA content (μmol g^−1^) decreased by 4.20%, 3.94%, 3.67% and 4.20%, respectively, compared to CK (*p* < 0.05).

In addition, H_2_O_2_ and MDA contents in *S. nigrum* shoots harvested at the first and second florescence stages were the lowest.

### 3.4. Effects of Different Types of N Fertilizers on Proline Concentration and the Activity of CAT, POD and SOD in S. nigrum Shoots

**Figure 1:**
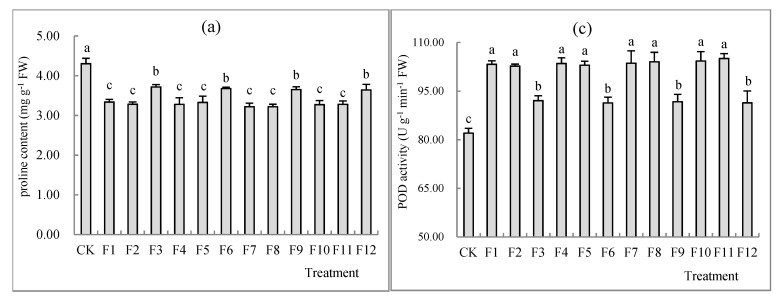

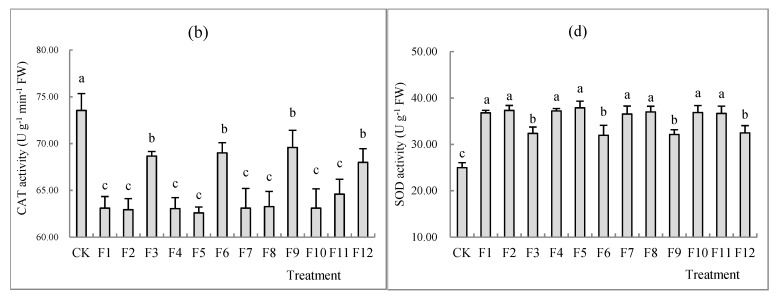
Effects of different types of N fertilizers on proline concentration (**a**) and the activity of CAT (**b**), POD (**c**) and SOD (**d**) by comparing single and double harvests of *S. nigrum* shoots (means with different letters in the panel are significantly different among treatments at *p* < 0.05. Error bars reported in figures are means of 3 replicates with standard deviation).

Figure 1a demonstrates that the addition of different N fertilizers ((NH_4_HCO_3_, NH_4_Cl, (NH_4_)_2_SO_4_ and urea (CH_4_N_2_O)) at different growth stages resulted in a significant decrease in antioxidant proline contents (mg g^−1^) and catalase (CAT) activities (U g^−1^ min^−1^) in *S. nigrum* shoots in comparison to CK with no fertilizer addition (*p* < 0.05); at the maturation stage, the proline contents (mg g^−1^) decreased by 13.63%, 14.56%, 15.10% and 15.34%, respectively, and CAT activities (U g^−1^ min^−1^) decreased by 6.64%, 6.17%, 5.38% and 7.54%, respectively, compared to CK (*p* < 0.05). Additionally, proline contents and CAT activities were the lowest at the first and second florescence stages (Figure 1a,b and Table 5).

At the same time, with the detection of another two types of enzymes, i.e., peroxidase (POD) and superoxide dismutase (SOD), the results show that after the application of different fertilizers, POD (U g^−1^ min^−1^) and SOD activities (U g^−1^) were markedly increased in comparison to CK with no fertilizer addition (*p* < 0.05); at the maturation stage, POD activities (U g^−1^ min^−1^) increased by 12.20%, 11.35%, 11.80% and 11.39%, respectively, and SOD activities (U g^−1^) increased by 29.55%, 27.95%, 28.62% and 29.95%, respectively (Figure 1c,d and Table 5).

In summary, the POD and SOD activities of the antioxidant system of *S. nigrum* with N fertilizers at the first and second florescence stages were higher than those at the maturation stage; however, the proline contents and CAT activities were diametrically opposite (Figure 1b–d and Table 5). The reason for the discrepancy was in relation to the harvesting mode, rather than fertilizer styles (Table 5).

### 3.5. Effects of Different Types of N Fertilizers on Extractable Cd Concentration in S. nigrum

**Figure 2:**
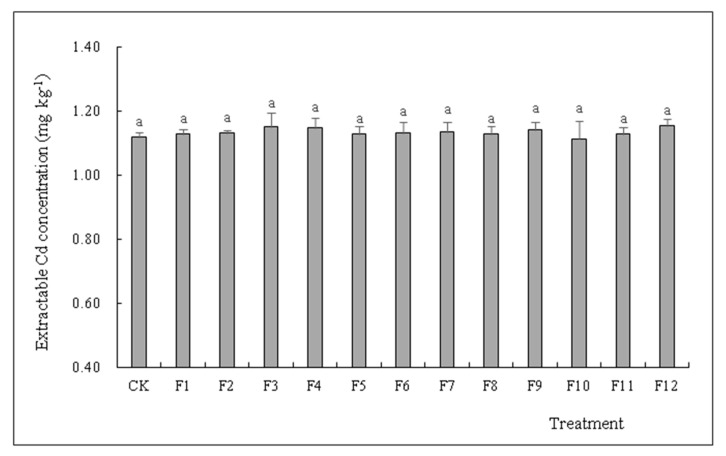
Effects of different types of N fertilizers on extractable Cd concentration by comparing single and double harvests of *S. nigrum* (means with different letters in the panel are significantly different among treatments at *p* < 0.05. Error bars reported in figures are means of 3 replicates with standard deviation).

As shown in Figure 2 and Table 5, the addition of four types of N fertilizers (NH_4_HCO_3_, NH_4_Cl, (NH_4_)_2_SO_4_ and urea (CH_4_N_2_O)) did not cause any significant difference in extractable Cd concentration in soil planted with *S. nigrum* harvested at different growing periods when compared to CK, and treatments were in the range of 1.11–1.15 mg∙kg^−1^ (*p* < 0.05).

### 3.6. Effects of Different Types of N Fertilizers on Soil pH in S. nigrum Cultivation

**Figure 3:**
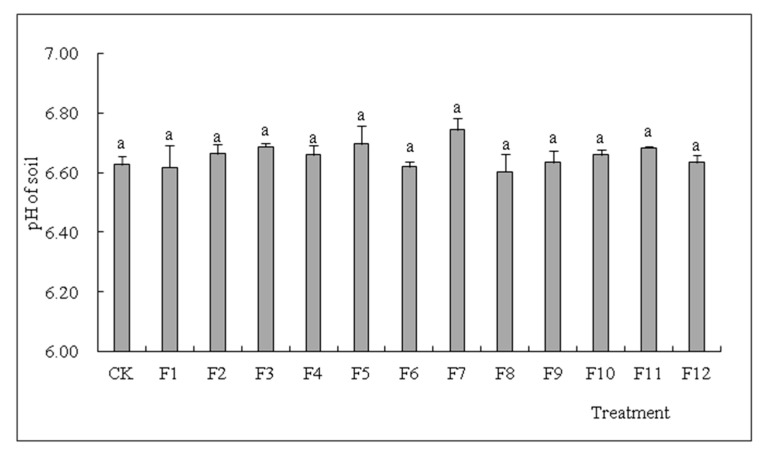
Effects of different types of N fertilizers on pH in soil as demonstrated by comparing single and double harvests of *S. nigrum.* (means with different letters in the panel are significantly different among treatments at *p* < 0.05. Error bars reported in figures are means of 3 replicates with standard deviation).

Comparing the one-stage and two-stage phytoremediation Cd experiments involving *S. nigrum*, the results show that the pH value in the rhizosphere soil used for *S. nigrum* cultivation under all N fertilizer treatment groups (NH_4_HCO_3,_ NH_4_Cl, (NH_4_)_2_SO_4_ and urea (CH_4_N_2_O) did not differ significantly compared to the control with no fertilizer supply and ranged from 6.60 to 6.70 (*p* < 0.05) (Figure 3 and Table 5).

## 4. Discussion

### 4.1. Effects of Different Fertilizers on S. nigrum Cd Phytoextraction in Relation to Single and Double Harvests

In a previous experiment, Wei et al. (2006) showed that the Cd hyperaccumulator *S. nigrum* could accumulate Cd from slightly-to-moderately Cd-contaminated soil by double harvesting during the growing season, thereby showing an enormous potential for improving Cd accumulation efficiency in practice [36], which is consistent with our finding. It was demonstrated that the addition of different N fertilizers significantly enhanced total Cd phytoremediation efficiency (μg plant^−1^) of *S. nigrum* under all treatments due to the significantly increased root and shoot biomasses (g plant^−1^) and maintenance of the Cd concentration in *S. nigrum* (Table 2, Table 3 and Table 5) and lead to higher shoot biomasses in double harvests compared to single harvests (Table 2 and Table 5). In contrast, other researchers conducted an experiment that demonstrated that NH_4_NO_3_ and Ca(H_2_PO_4_)_2_ application did not increase biomass, while Cd concentrations significantly decreased compared to unfertilized plants [27]. These authors concluded that the reason was the formation of metal phosphate chelate, which reduced the solubility and mobility of heavy metal in soil. Similar to the above results, Fässler et al. (2010) also reported that (NH_4_)_2_SO_4_ had no obvious effect on Cd accumulation by maize [37].

However, other pot culture experiments showed that N fertilizer significantly improved the biomass and Cd phytoremediation efficiency of *Tagetes patula* grown in Cd-contaminated soil [38]. Nitrogen supply, particularly nitrate, sharply increased Cd extraction of *T. caerulescens* [39], while urea significantly enhanced Cd phytoextraction by *Carpobrotus rossii* [40], which further demonstrated that the addition of N fertilizer to soil plays an important role in Cd phytoaccumulation efficiency. Moreover, different types of N fertilizers exerted various effects on different plant species [39,41].

### 4.2. Effects of Different Fertilizers on H_2_O_2_, MDA and Proline Contents and Antioxidant Enzyme Activities in S. nigrum in Relation to Single and Successive Harvests

It has been confirmed that N fertilizers effectively increase plant biomass by supplementing required nutrients, while enhancing the plant antioxidant system composed of enzymatic and non-enzymatic antioxidants. This in turn improves the resistance and tolerance of plants exposed to Cd stress [42,43], which corresponds to another study on the hyperaccumulator *Brassica juncea* grown in contaminated sediments; the research showed that N fertilizer not only increases boron phytoextraction, but also alleviates B stress [44]. Huang et al. (2019) reported that mineral fertilizers reduced the content of H_2_O_2_ and MDA in wheat [45]. H_2_O_2_ and MDA are the major oxidation products of lipid peroxidation and can directly reflect the degree of oxidative stress. Accordingly, in our pot culture experiment, it was discovered that after applying nitrogen fertilizer, the contents of H_2_O_2_ and MDA in *S. nigrum* shoots at the maturation stage effectively decreased compared to CK (*p* < 0.05) (Table 3 and Table 5), which revealed that N fertilizers could remarkably strengthen the stress tolerance and promote the plant growth.

As one of the most common low-weight organic solutes, proline has a vital role in stabilizing protein complexes and scavenging free radicals in an antioxidative system. It has been demonstrated that endogenous proline is overproduced in many plant species when subjected to abiotic and biotic stresses, and the proline accumulation was closely related to H_2_O_2_ generation under stress conditions [46]. This is consistent with the results of our study, which reveal that the H_2_O_2_ and proline concentration were considerably decreased after N treatment in *S. nigrum* shoots in different growth stages (Table 3 and Table 5 and Figure 1a). Moreover, some other studies showed that, in adversity circumstances, antioxidant defense mechanisms were activated and antioxidant enzymes activities were enhanced. SOD, when combined with POD and CAT, is considered as the first defensive barrier of the organisms and exerts a synergistic effect on ROS scavenging in which SOD converts O_2_^−^ to H_2_O_2_ and oxygen, whereafter CAT and POD participate in scavenging, converting H_2_O_2_ to O_2_ to relieve the stress [47]. In our present study, it was found that CAT activity was considerably decreased in *S. nigrum* shoots at different growth stages; in contrast, POD as well as SOD activities increased (Figure 1b–d and Table 5). Our conclusion is similar to the conclusions of Sun et al. (2015), who discovered that POD activity was elevated after fertilizer application [48]. On the contrary, Huang et al. (2019) found decreased SOD activity after fertilizer application [45] was correlated with many influence factors, such as plant species, fertilizer quantity and types, Cd concentrations and exposure times [49]. Furthermore, SOD not only regulates the oxidative stress, but also plays an important role in the whole process of growth, development and reproduction in plants. In another study on two species of wheats, it was concluded that after the application of Si fertilizer, the antioxidant activity in a Cd-tolerant cultivar remarkably improved compared to that in a Cd-sensitive cultivar [50], which proves that antioxidant enzyme activities mainly depend on genotypic specificity.

In addition, POD and SOD activities in *S. nigrum* shoots reached the maximum at the first and second florescence stages (Figure 1c,d), which is consistent with the results of Esmaeili et al. (2018), who reported that the antioxidant activity of *Oliveria decumbens* Vent. (Apiaceae) was the highest during flowering stages [51].

### 4.3. Effects of Different Fertilizers on S. nigrum Extractable Cd Concentration in Relation to Single and Double Harvests

Our study showed that different types of N fertilizers did not significantly increase extractable Cd concentration compared to CK (*p* < 0.05) (Figure 2 and Table 5). Generally, Cd availability and solubility affects extractable Cd concentration in the soil, and variation in Cd solubility depends on the pH value and organic matter content in soil [38].

### 4.4. Effects of Different Fertilizers on the pH Value in Comparison to Single and Double Harvests of S. nigrum

Some articles have reported that after ammonium fertilizer soil application, the pH decrease in the soil might be attributed to NH^4+^ ion exchange and assimilation [52]. However, in our study, after the application of N fertilizer, the pH value in the soil showed no significant change compared to CK without N fertilizer addition (*p* < 0.05) (Figure 3 and Table 5). Nevertheless, only physicochemical conditions were taken into account from the above conclusions. In addition, root exudates and metabolites released by growing plants and some microorganisms in the soil would regulate soil pH, so that plants could grow normally and healthily in the available root zone environment [2,53].

## 5. Conclusions

Cd phytoremediation efficiency of *S. nigrum* improved with the addition of N fertilizers (NH_4_HCO_3_, NH_4_Cl, (NH_4_)_2_SO_4_, CH_4_N_2_O) (*p* < 0.05); moreover, the Cd phytoaccumulation capacity of *S. nigrum* was higher during double harvests compared to single harvests due to the increase in *S. nigrum* shoot biomass and no obvious changes in Cd concentration. The pH value and extractable Cd content did not show a significant variation trend. In addition, by analyzing the content and activity of non- and enzymatic antioxidants, it was found that compared to the single-cropping system, *S. nigrum* grew better after fertilization, and showed the highest efficiency in terms of Cd phytoremediation when harvested using a double-cropping system. The differences were associated with the harvesting mode, rather than fertilizer styles.

## Figures and Tables

**Table 1:** Experimental treatment with 4 kinds of nitrogen fertilizers.

No.	Treatment	Dose (g∙kg^−1^)	Added Total N (mg∙kg^−1^)	Harvest Time
CK	Control, no N addition	0.00	0.00	at maturation stage
F1	NH_4_HCO_3_	1.68	300.00	at the first florescence stage
F2	NH_4_HCO_3_	1.68	300.00	at the second florescence stage
F3	NH_4_HCO_3_	3.36	600.00	at maturation stage
F4	NH_4_Cl	1.14	300.00	at the first florescence stage
F5	NH_4_Cl	1.14	300.00	at the second florescence stage
F6	NH_4_Cl	2.28	600.00	at maturation stage
F7	(NH_4_)_2_SO_4_	1.41	300.00	at the first florescence stage
F8	(NH_4_)_2_SO_4_	1.41	300.00	at the second florescence stage
F9	(NH_4_)_2_SO_4_	2.82	600.00	at maturation stage
F10	CH_4_N_2_O	0.65	300.00	at the first florescence stage
F11	CH_4_N_2_O	0.65	300.00	at the second florescence stage
F12	CH_4_N_2_O	1.30	600.00	at maturation stage

Note: doses of inorganic compounds are supplemented for analytically pure reagents.

**Table 2:** Effects of different types of N fertilizers on Cd phytoextraction in *S. nigrum*.

Treatment	Roots (mg∙kg^−1^)	Shoots (mg∙kg^−1^)	Shoot Cd Extraction (μg∙Plant^−1^)
CK	20.11 ± 0.55 a	20.61 ± 0.63 a	17.66 ± 0.57 e
F1	19.66 ± 0.37 a	20.38 ± 0.31 a	25.29 ± 0.56 d
F2	19.94 ± 0.66 a	21.05 ± 0.26 a	23.03 ± 0.49 d
F3	19.53 ± 0.24 a	21.08 ± 0.25 a	38.86 ± 0.58 b
F4	20.27 ± 0.42 a	21.00 ± 0.45 a	27.01 ± 0.68 d
F5	20.65 ± 0.27 a	21.05 ± 0.23 a	24.21 ± 0.28 d
F6	19.73 ± 0.41 a	20.27 ± 0.34 a	37.76 ± 0.79 b
F7	20.35 ± 0.68 a	21.09 ± 0.21 a	34.41 ± 0.30 c
F8	19.82 ± 0.41 a	21.00 ± 0.30 a	33.61 ± 0.39 c
F9	19.81 ± 0.49 a	21.07 ± 0.05 a	50.83 ± 0.55 a
F10	20.26 ± 0.75 a	21.35 ± 0.32 a	33.54 ± 0.33 c
F11	19.75 ± 0.58 a	20.80 ± 0.23 a	33.06 ± 0.35 c
F12	19.92 ± 0.24 a	20.84 ± 0.48 a	50.16 ± 1.02 a

Note: Means of 3 replicates with standard deviation followed by the same letter within the same column are not significantly different (*p* < 0.05).

**Table 3:** Effects of different types of N fertilizers on *S. nigrum* root and shoot biomasses (g plant^−1^).

Treatment	Roots (g∙Plant^−1^)	Shoots (g∙Plant^−1^)
CK	0.16 ± 0.01 e	0.86 ± 0.02 e
F1	0.37 ± 0.01 cd	1.24 ± 0.02 d
F2	0.35 ± 0.01 d	1.09 ± 0.01 d
F3	0.41 ± 0.01 c	1.84 ± 0.02 b
F4	0.39 ± 0.02 c	1.29 ± 0.01 d
F5	0.37 ± 0.02 cd	1.15 ± 0.01 d
F6	0.42 ± 0.01 c	1.86 ± 0.01 b
F7	0.61 ± 0.01 b	1.63 ± 0.03 c
F8	0.57 ± 0.01 b	1.60 ± 0.03 c
F9	0.69 ± 0.02 a	2.41 ± 0.01 a
F10	0.61 ± 0.01 b	1.59 ± 0.02 c
F11	0.58 ± 0.02 b	1.59 ± 0.02 c
F12	0.70 ± 0.01 a	2.41 ± 0.02 a

Note: means of 3 replicates with standard deviation followed by the same letter within the same column are not significantly different (*p* < 0.05).

**Table 4:** Effects of different types of N fertilizers on H_2_O_2_ amd MDA in *S. nigrum* shoots.

Treatment	H_2_O_2_ (mg g^−1^ FW)	MDA (μmol g^−1^ FW)
CK	0.46 ± 0.03 a	6.27 ± 0.04 a
F1	0.31 ± 0.02 c	5.57 ± 0.03 c
F2	0.32 ± 0.02 c	5.55 ± 0.03 c
F3	0.39 ± 0.03 b	6.00 ± 0.05 b
F4	0.31 ± 0.01 c	5.51 ± 0.02 c
F5	0.30 ± 0.02 c	5.53 ± 0.03 c
F6	0.38 ± 0.02 b	6.02 ± 0.07 b
F7	0.33 ± 0.03 c	5.50 ± 0.03 c
F8	0.29 ± 0.02 c	5.52 ± 0.04 c
F9	0.37 ± 0.02 b	6.04 ± 0.04 b
F10	0.30 ± 0.01 c	5.49 ± 0.04 c
F11	0.34 ± 0.02 c	5.52 ± 0.04 c
F12	0.38 ± 0.04 b	6.03 ± 0.05 b

Note: means of 3 replicates with standard deviation followed by the same letter within the same column are not significantly different (*p* < 0.05).

**Table 5:** The two-way variance analysis of Cd phytoremediation and biochemical indicators in *S. nigrum* under different fertilizer types and harvesting mode treatments.

Treatment	Root (mg∙kg^−1^)	Shoot (mg∙kg^−1^)	Shoot Extraction (μg∙Plant^−1^)	Root (g∙Plant^−1^)	Shoot (g∙Plant^−1^)	H_2_O_2_ (mg∙g^−1^)	MDA (μmol∙g^−1^)	Proline (mg∙g^−1^)	CAT (U g^−1^∙min^−1^)	POD (U g^−1^∙min^−1^)	SOD (U∙g^−1^)	Extractable Cd Concentration (mg∙kg^−1^)	pH Value of Soil
F (FT)	1.609	1.526	914.06 *	1324.7 *	1579.7 *	0.325	1.404	0.335	0.212	0.329	0.18	0.035	0.021
F (HM)	2.03	0.884	2852.6 *	166.4 *	5376.2 *	43.5 *	663.2 *	101.2 *	58.9 *	109.7 *	48.5 *	0.926	1.374
F (FT × HM)	1.061	1.988	18.29 *	8.048 *	27.3 *	0.966	1.015	0.15	0.746	0.237	0.211	0.472	1.957

Note: means of 3 replicates with standard deviation with different letters in the column are significantly different at *p* < 0.05. F: F test. * is significant difference at *p* < 0.05. FT: fertilizer type. HM: harvesting mode. FT × HM: fertilizer type × harvesting mode. (df(FT) = 3; df(HM) = 2; df(FT × HM) = 6). F(FT): F (value) (fertilizer type); F(HM): F (value) (harvesting mode); F(FT × HM): F(value) (fertilizer type × harvesting mode).

## Data Availability

The datasets used or analyzed during the current study are available from the corresponding author on reasonable request.
